# Coping strategies facing Covid-19, perceived social support, and trait anxiety among Tunisian caregivers

**DOI:** 10.1192/j.eurpsy.2024.1053

**Published:** 2024-08-27

**Authors:** N. Smaoui, B. Jallouli, I. Gassara, R. Feki, S. Omri, M. Bou Ali Maalej, N. Charfi, J. Ben Thabet, L. Zouari, M. Maalej

**Affiliations:** ^1^Psychiatry C department, Hedi Chaker university hospital; ^2^Faculty of Medicine, Sfax; ^3^University of Sfax, SFAX, Tunisia

## Abstract

**Introduction:**

Caregivers in the Sfax region, Tunisia, having been at the forefront in the face of the Covid-19 pandemic, were therefore faced with intense stress. It seemed useful and interesting to us to study their adaptation strategies during this period of pandemic.

**Objectives:**

The aims of our study were to identify the coping strategies used by Tunisian Healthcare workers (HCW) during the Covid-19 pandemic and to study the links of the different coping strategies with perceived social support and trait anxiety.

**Methods:**

A cross-sectional, descriptive, and analytical study conducted among 254 Tunisian HCW working at the Habib Bourguiba and Hedi Chaker university hospitals in Sfax, during period from January 2021 to April 2021. the questionnaire used included an information sheet and three scales; “Social support questionary 6” (SSQ-6), “State Trait Inventory Anxiety Form Y2” (STAI-Y2), and “Ways of Coping Checklist” (WCC).

**Results:**

Using the WCC scale, the strategy most used by participants was the problem-focused one (M = 2.98 ±0.53), followed by the emotion-focused strategy (M = 2.65 ±0 .58), and that centered on the search for social support (M = 2.64 ±0.59). Using the SSQ-6, the mean score for the availability of perceived social support was equal to 8.91±4.59 and the score mean perceived satisfaction was equal to 28.63±5.84. The prevalence of trait anxiety was 50%, according to the STAI-Y2. Statistical tests showed that problem-focused coping was the strategy most adopted by non-anxious participants. They also showed that the higher the availability of perceived social support, the more the social support-seeking coping strategy was chosen, and the higher the perceived satisfaction with perceived social support, the less the emotion-focused strategy was chosen.

**Conclusions:**

It seems necessary to propose a learning program for coping strategies to counter the potential emergence of ineffective strategies and to reinforce the use of effective strategies, in order to improve or maintain optimal well-being of health personnel.

**Disclosure of Interest:**

None Declared

